# Effects of protective factors on the depressive status of elderly people in Taiwan

**DOI:** 10.1097/MD.0000000000018461

**Published:** 2020-01-03

**Authors:** Yang-Tzu Li, Tao-Hsin Tung

**Affiliations:** a National Taipei University of Nursing and Health Science; b Department of Medical Research and Education, Cheng Hsin General Hospital, Taipei, Taiwan.

**Keywords:** crisis episodes, depression, protective factors, resilience in the elderly people, social participation, social support

## Abstract

This study was conducted to examine the effect of protective factors on the relationship between crisis episodes and depression in the elderly population in Taiwan.

In this study, the Taiwan Longitudinal Study on Aging was used as basis for a cross-sectional secondary data analysis. After eliminating respondents below the age of 65 years and those with missing values, 2426 samples were collected. Predictive variables, such as crisis episodes, personal resources, family ties, social participation, and social support, were investigated, and the dependent variable of “depression status” was measured using the Center for Epidemiologic Studies Depression scale.

According to the results of regression analysis, the protective factors of self-assessed health (ß = −0.290, *P* < .001), instrumental support (ß = −0.153, *P* < .001), financial satisfaction (ß = −0.126, *P* < .001), emotional support (ß = −0.101, *P* < .001), crisis episodes (ß = 0.087, *P* < .001), support satisfaction (ß = −0.081, *P* < .001), leisure participation (ß = −0.053, *P* < .05), family ties (ß = −0.048, *P* < .05), and community participation (ß = −0.042, *P* < .05) had a significant effect on depression status. Moreover, leisure participation had a moderating effect on the relationship between crisis episodes and depression (ß = −0.07, *P* < .01). In addition, according to path analysis results, family ties had a significant negative predictive power on depression (β = −0.225, *P* < .001), as did social support (β = −0.978, *P* < .001). The predictive power of crisis episodes on depression through social support was 0.197 (−0.201 × −0.978 = 0.197, *P* < .001), and it was −0.324 (−0.331 × −0.978 = −0.324, *P* < .001) through social participation, which indicated that social support plays a mediating role between crisis episodes and depression and between social participation and depression.

Strengthening effective protective factors can improve the resilience of elderly people and enable them to cope with dilemmas rapidly and effectively when faced with crisis episodes as well as restore their health status and enjoy a satisfactory life.

## 1 Introduction

In the context of major Asian countries, the proportion of elderly people in Taiwan is second only to that in Japan (28%) and comparable to that in South Korea (14%).^[1]^ According to the National Development Council of the Executive Yuan, the proportion of elderly people will reach 20% in 2025, making Taiwan a “super-aged” society. Taiwan's transition from an aged to a super-aged society will have taken only 7 years, which is faster than estimates for Japan (11 years), the United States (14 years), France (29 years), and the United Kingdom (51 years) and comparable to estimates for South Korea (8 years) and Singapore (7 years),^[2]^ reflecting the severity of the aging process. Studies have indicated that elderly people often experience more crisis episodes than people at other stages of life. These crisis episodes include retirement, bereavement, and multiple diseases,^[3,4]^ which can result in the collapse of one's problem-solving abilities or lead to the adoption of ineffective coping strategies.^[5]^

Crisis episodes may increase depressive symptoms in elderly people,^[6–8]^ possibly leading to suicidal ideation and even suicide.^[9]^ Several studies have indicated that some individual and environmental factors can aid in the maintenance of positive adaptation in adversity. These psychological and social resources are termed “protective factors.”^[10]^ Protective factors enable resilience to carry out its function. Academic interest in resilience in the elderly population is driven by positive psychology to a certain extent.^[11]^ Presently, the focus of research on resilience has changed from the examination of negative emotions or abnormal psychology to that of positive psychology.^[12,13]^ Richardson^[14]^ observed that studies on resilience have gradually shifted their focus from identifying problems initially to increasing the strength of coping strategies and crisis prevention presently. Such studies emphasize protective factors, such as individual potential, family support, and environmental resources, which can alleviate the effect of crisis episodes.

Several previous studies have identified the protective mechanism of resilience as part of one's resources and capabilities. Subsequently, the focus on family resources^[7]^ and external environmental resources, such as social participation and social support,^[8]^ has gradually increased, and some valuable conclusions have been derived. However, few studies have simultaneously examined the combined effects of individual, familial, and environmental protective factors on the positive adaptation of elderly people who encounter crisis episodes. This study examined the effects of crisis episodes on depression among elderly Taiwanese people. In addition, this study analyzed the predictive power of one's resources, family ties, social participation, and social support protective factors on depression. Finally, the moderating and mediating effects of protective factors on the relationship between crisis episodes and depression were evaluated.

## 2 Methods

### 2.1 Data source

In the present study, the 2011 Taiwan Longitudinal Study on Aging (TLSA) was used as basis for a secondary data analysis. This database was established by the Institute of Family Planning in 1989. The data it contains were collected using a long-term panel study design with a completion rate and follow-up of migrated cases over 80%. Sampled participants were middle-aged to elderly people from more than 300 townships and urban districts in Taiwan. Three-segment probability proportional to size sampling was used. Township and urban district samples were selected based on equal probabilities before the selection of neighborhood samples. Finally, 2 people from each neighborhood were selected as survey participants. An onsite survey was performed in October 2011, and a total of 3727 survey samples were collected over an 8-month period. After eliminating respondents below the age of 65 years and those with missing values, a total of 2426 samples were collected for analysis.

### 2.2 Study variables

Depression status in elderly people is related to crisis episodes and protective factors. The association between crisis episodes and depression may be affected by the presence of protective factors. Therefore, the following hypotheses were proposed to be examined in this study. Hypothesis 1: The higher the number of crisis episodes experienced, the more severe the depression. Hypothesis 2: Protective factors have a significant predictive power in alleviating depression. Hypothesis 3: Protective factors have significant moderating and mediating effects on the association between crisis episodes and depression.

The study variables were as follows:

1. Dependent variable: The dependent variable was depression status. In this study, the shortened Center for Epidemiologic Studies Depression (CES-D) scale, which was applied in the 2011 TLSA, was used to measure depression status in elderly people. The CES-D scale was originally developed by Radloff in 1977 as a self-report scale for depressive symptoms in the general population with references to scales with established good validity and reliability, such as the Zung Self-Rating Depression Scale, Beck Depression Inventory, and Minnesota Multiphasic Personality Inventory. The original scale contained 20 questions covering the symptoms of depressed mood, guilt and worthlessness, feelings of helplessness and hopelessness, psychomotor retardation, loss of appetite, and sleep disturbance. Given that a healthy elderly person takes approximately 7 minutes to complete the original scale and that an elderly person with poor physical health may require up to 12 minutes, some researchers selected questions with a higher factor load to shorten the overall interview time and reduce the burden on elderly respondents, which in turn reduced the number of CES-D questions.^[15]^ The TLSA employed the 11-question version of the CES-D scale developed by Kohout et al.^[15]^ Elderly people in the United States were used as the sample population, and the internal consistency coefficient ranged between 0.76 and 0.81.^[15]^ Jou and Chuang^[16]^ applied this scale to elderly people in Taiwan in 1989 and 1993, and the correlation coefficient of total scores in the 2 cohorts was 0.4. Questions regarding depression status covered the following aspects: did not feel like eating, poor appetite; felt that everything required effort; restless sleep; felt in a bad mood; felt lonely; felt people around were not friendly (unfriendly, cold); felt sad; could not get “going;” felt happy; enjoyed life; and felt that people around disliked me. The result was based on the mean score of all questions. The higher the score, the more severe the depressive status.
2. Independent variables: Independent variables were crisis episodes, self-assessed health, activity function, financial satisfaction, family ties, community participation, leisure participation, emotional support, instrumental support, and support satisfaction. Questions for measuring crisis episodes included the death of pets, death of close family members (not including spouse), death of close friends, reduced income, investment or credit difficulties, inability to pay mortgage or other loans, poor health or behavior of relatives, poor marital relationship, loss of personal assets, serious quarrels with friends or family, retirement, and serious injury or illness. The result was obtained through the summation of all items’ scores. The higher the score, the more severe the crisis episode.

The self-assessed health-related question was “How do you feel about your current health status?” The higher the score, the better the self-assessed health. The Activities of Daily Living scale was used for questions on activity function. The 6 main activities of daily living that were measured were bathing, wearing clothes, eating, getting out of bed, walking indoors, and going to the washroom. The result was based on the average score of the 6 items. The higher the score, the better the physical function. The financial satisfaction-related question was “Overall, are you satisfied with your current financial status?” The higher the score, the greater the satisfaction with one's financial status. The question on family ties dealt with the number of relatives residing with the participant.

Community participation items included questions on participation in friendship groups; trade unions for agricultural, fishing, or other industry; political groups; social service associations; clans; elderly groups; and elderly learning activities. The result was obtained by the summation of scores for participating in various activities. Leisure participation was assessed based on 14 activities among which playing chess or card games, chatting with friends and family, or brewing “Kung Fu Tea” (one type of cuncentrated Chinese tea). The higher the score, the greater the social participation in various items.

Emotional support-related questions were “When you need to discuss problems or worries with others, do you feel that your family members or friends are willing to listen to you?” and “Do you feel your family members or friends care about you?” The final score was the average of these 2 questions’ scores. The instrumental support question was “Can you rely on your family members and friends when you are sick or require care?” The support satisfaction question was “Are you generally satisfied with the level of concern shown by your family members and friends?” The higher the score, the greater the social support.

### 2.3 Data availability

The approval number for this study is NTU-REC No. 201805ES040. The [S_BHP_TWELD100.excl] data used to support the findings of this study were supplied by the Ministry of Health and Welfare Data Science Center under license. Therefore, they cannot be made freely available. Requests for access to these data should be made to the Ministry of Health and Welfare Data Science Center [https://dep.mohw.gov.tw/ DOS/np-2503–113.html]. In addition, the [S_BHP_TWELD100.excl] data used to support the findings of this study may be released upon application to the Ministry of Health and Welfare Data Science Center, which can be contacted at https://dep.mohw.gov.tw/DOS/np-2503–113.html.

### 2.4 Statistical analysis

The SPSS statistical software suite (IBM Corp., New York, NY) was used for analysis in this study. Variables related to the study were recoded to match the study design. Descriptive statistics are presented as the number of participants and percentages to understand the distribution of sex, age, educational level, and marital status in elderly participants. A variance analysis was conducted to examine differences in depression based on sex, age, educational level, and marital status. An explanatory multiple hierarchical regression analysis was performed to predict the dependent variable based on the independent variables. Two independent variables, namely individual attributes and crisis episodes, were included in the regression analysis model 1. Protective factors were added to model 2 to understand the predictive power of protective factors when crisis episodes were encountered. Interaction variables between crisis episodes and protective factors were added in model 3 to understand the moderating effects of protective factors on the association between crisis episodes and depression.

To examine the effect paths of crisis episodes, family ties, social participation, and social support on depression, leisure and community participation were combined into “social participation” and emotional support, instrumental support, and support satisfaction were combined into “social support.” The AMOS statistical software (IMB Corp, Amonk, New york) package was used to conduct a structural equation modeling path analysis with the maximum likelihood method. In addition, the chi-square test, root mean square error of approximation (RMSEA), goodness of fit index (GFI), comparative fit index (CFI), and standardized root mean square residual (SRMR) were used to determine the fit of the model. Because the chi-square value (χ^2^) can become overly sensitive when the sample size is large, it was listed only as a reference. The closer the GFI is to 1, the better the model fit. The smaller the RMSEA value, the more ideal the fit of the model, and a model with an RMSEA value of <0.06 can be considered a good model. The CFI value reflects the difference between the hypothetical model and the independent model with no covariate relationships. The closer the CFI is to 1, the more ideal the model. When the SRMR is lower than 0.08, the residual is low, and model fit is thus good.

## 3 Results

### 3.1 Analysis of sample basic characteristics

In total, 2426 valid samples were collected in this study. The sex ratio of participants was relatively similar but with a few more female participants (51.3%). Participants were divided into 4 age groups, namely 65 to 69, 70 to 74, 75 to 79, and ≥80 years. At 38.9%, participants ≥80 years of age accounted for most of the study population. Most participants were married, and their spouses were still alive (55.8%). Widows and widowers who did not remarry accounted for 39.6% of the study population, suggesting that the spouses of most elderly people were still alive. The educational level of participants was divided into 4 categories, and those with an educational level of elementary school and below formed the majority (75.7%), followed by senior and vocational schools (11.4%) (Table 1).

**Table 1:**
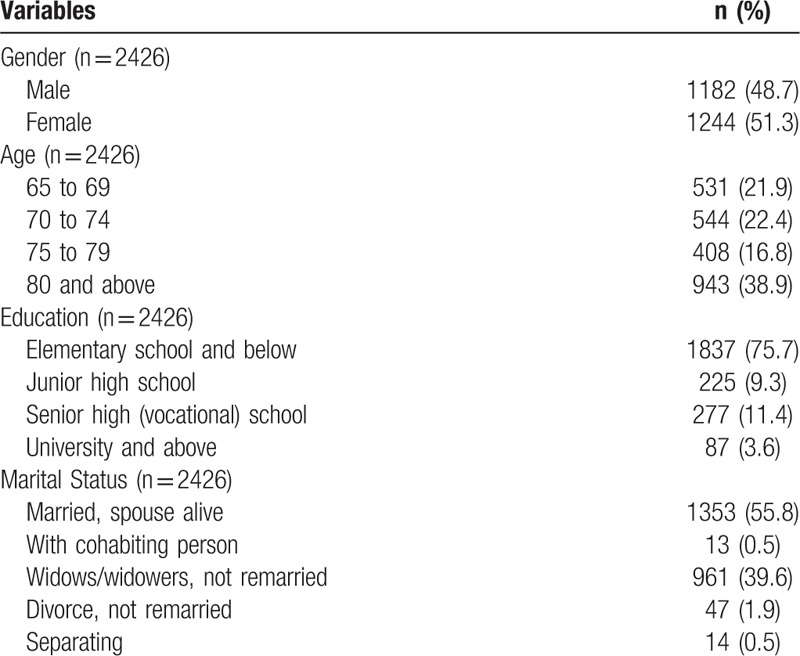
Distribution of basic characteristics.

### 3.2 Variance analysis of the dependent variable based on attribute variables

Table 2 indicates that female participants had a higher mean depression score than did male participants. According to the results of one-way ANOVA, the age groups exhibited significant differences in depression (F = 8.672, *P* < .001). Scheffe post hoc comparison revealed that depression scores in elderly people aged 70 to 74, 75 to 79, and ≥80 years were higher than those in elderly people aged 65 to 69-years. There were also significant differences in depression between participants according to their marital status (F = 16.466, *P* < .001). Scheffe post hoc comparison revealed that depression scores in elderly widows or widowers who did not remarry were higher than those in elderly people whose spouses were still alive. An analysis of educational level groups indicated that there were significant differences in depression between participants according to their educational level (F = 10.134, *P* < .001). Scheffe post hoc comparison revealed that depression scores in elderly people who had an educational level of elementary school and below were higher than those in elderly people with an educational level of high school (vocational school) and university or above.

**Table 2:**
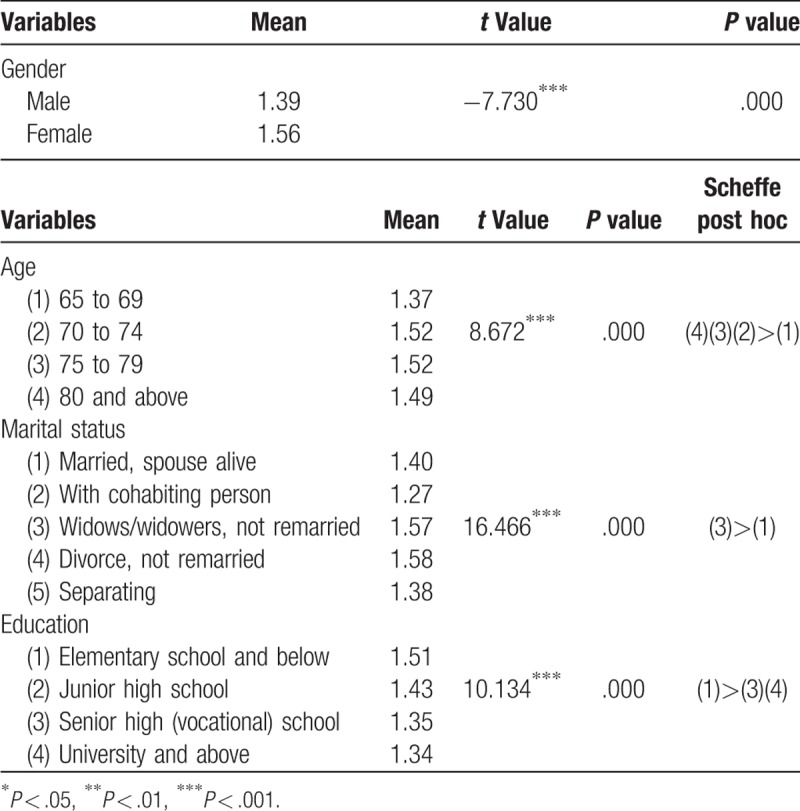
*t* Test and variance analysis in depression by individual attributes.

### 3.3 Analysis of samples with high and low depression status

A total of 858 participants with depression scores >1.45 points (66.7 percentile) were categorized as samples with high depression status, and 885 participants with depression scores <1.18 points (33.3 percentile) were categorized as samples with low depression status. These 1743 samples in total were categorized into people who experienced crisis episodes with a high depression status, experienced crisis episodes with a low depression status, had not experienced crisis episodes with a high depression status, and had not experienced crisis episodes with a low depression status. Table 3 illustrates that there were significant differences in protective factor variables among these 4 groups including self-assessed health, activity function, financial satisfaction, family ties, community participation, leisure participation, emotional support, instrumental support, and support satisfaction (all *P* < .001). Post hoc comparison using Scheffe method indicated that elderly people with higher scores in various protective factors had a significantly lower level of depression than did those with lower scores in various protective factors, regardless of whether they had experienced crisis episodes. Therefore, these protective factors were proved to have protective effects on elderly people who experienced crisis episodes. The results demonstrated that self-assessed health, activity function, financial satisfaction, family ties, community participation, leisure participation, emotional support, instrumental support, and support satisfaction can be major assets to reduce depression in elderly people who experience crisis episodes.

**Table 3:**
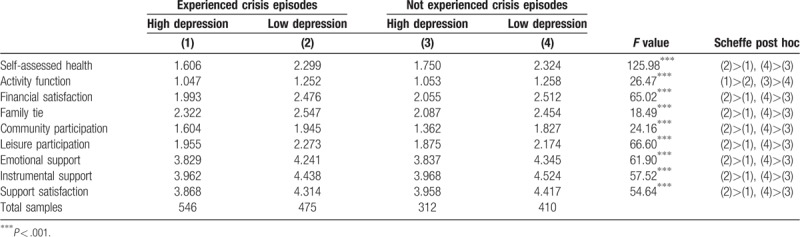
The effect of protective factors between high and low depression groups.

### 3.4 Predictive model of protective factors in elderly people

Multiple hierarchical regression analysis was conducted to elucidate and compare relationships between variables. The explanatory power of the overall regression model (*R*^2^) was reported using the *F* test of significance. Once this was deemed significant, individual explanatory power testing of various independent variables was performed, followed by a comparison of their relative importance. The analysis required converting categorical variables into dummy variables. Among these variables, male sex was used as the control group for sex, the age group from 65 to 69 years was used as the control group for age, the group of married and living spouses was used as the control group for marital status, and an educational level of elementary school and below was used as the control group for educational level.

Three models were used for testing protective factors in elderly people. In model 1, only individual attributes that were considered as control variables together with crisis episode variables were set as dependent variables. In model 2, protective factors were added. In model 3, interaction variables obtained from the product of protective factors and crisis episodes analysis were added.

Regression analysis demonstrated significant differences in the overall regression modes of the 3 models (*F* values of 17.845, 53.948, and 37.791; *P* < .001). This implied that at least 1 predictive variable in the 3 models reached significance. In the regression analysis of model 1, individual attribute variables of elderly people and crisis episodes were included, which explained 7.9% of the variance in depression. Sex, age, marital status, and crisis episodes were the main predictive factors. In model 2, protective factor variables were added to the regression analysis, and the explanatory power of the entire prediction was 33.0%, which was far higher than the predictive power of model 1 (7.9%). Additionally, the coefficient for crisis episodes decreased from 0.182 to 0.087, demonstrating the importance of protective factors in the psychological health of elderly people. When comparing standardized regression coefficients, regarding predictor variables that affect depression, self-assessed health was the most important (ß = −0.290, *P* < .001), followed by instrumental support (ß = −0.153, *P* < .001), financial satisfaction (ß = −0.126, *P* < .001), emotional support (ß = −0.101, *P* < .001), crisis episodes (ß = 0.087, *P* < .001), support satisfaction (ß = −0.081, *P* < .001), leisure participation (ß = −0.053, *P* < .05), family ties (ß = −0.048, *P* < .05), and community participation (ß = −0.042, *P* < .05).

In model 2, protective factors were added. Elderly people with poorer self-assessed health, dissatisfaction with their financial status, poorer emotional support, support satisfaction, and instrumental support, lower leisure participation, fewer family ties, and lower community participation were more likely to develop depression. In addition, the greater the number of crisis episodes experienced, the more likely were the depressive symptoms.

In model 3, the products of protective factor variables (self-assessed health, activity function, financial satisfaction, family ties, community participation, leisure participation, emotional support, instrumental support, and support satisfaction) and crisis episodes were included in the regression model, which had an explanatory power of 33.2% for depression. The overall explanatory power increased by 0.2% compared with that of model 2. This implied that interaction items slightly improved the fit of the overall regression model. Among these interactions, crisis episodes × leisure participation was significant, suggesting that leisure participation had a moderating effect on the relationship between crisis episodes and depression (ß = −0.07, *P* < .01), which also indicated that leisure participation can reduce the effects of crisis episodes on depression.

**Figure 1:**
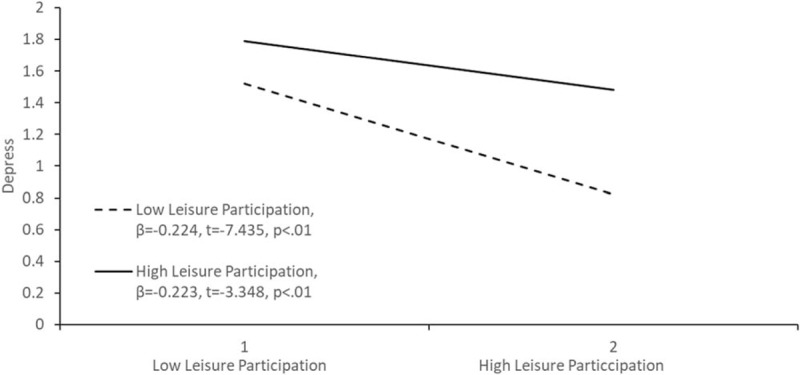
. Moderating effects of leisure participation on the relationship between crisis episodes and depression.

To understand the type of moderating effect of leisure participation, 1 standard deviation was added or subtracted to the mean scores of crisis episodes and leisure participation to form high- and low-score groups. Analysis results indicated that depression was alleviated when leisure participation was high, regardless of crisis episodes in high- or low-score groups. In particular, depressive emotions were drastically decreased by an increase in leisure participation in elderly people who had experienced fewer crisis episodes (Fig. 1).

### 3.5 Structural equation modeling path analysis

The path model constructed in this study was proved to have good goodness of fit, with χ^2^ = 6.59 and *P* < .001, reaching significance. This indicated that the null hypothesis was true. RMSEA was 0.018, lower than the threshold of 0.05, suggesting that the saturated model difference between the theoretical model and complete fit was low. SRMR was 0.028, which was lower than the threshold of 0.08, indicating that the residual was small. The CFI was 0.955, and GFI was 0.996, which was higher than the threshold of 0.90. Overall, the goodness of fit of the path model in this study was relatively ideal. Residual analysis and inspection of the model correction index revealed that this model did not require additional modifications.

**Figure 2:**
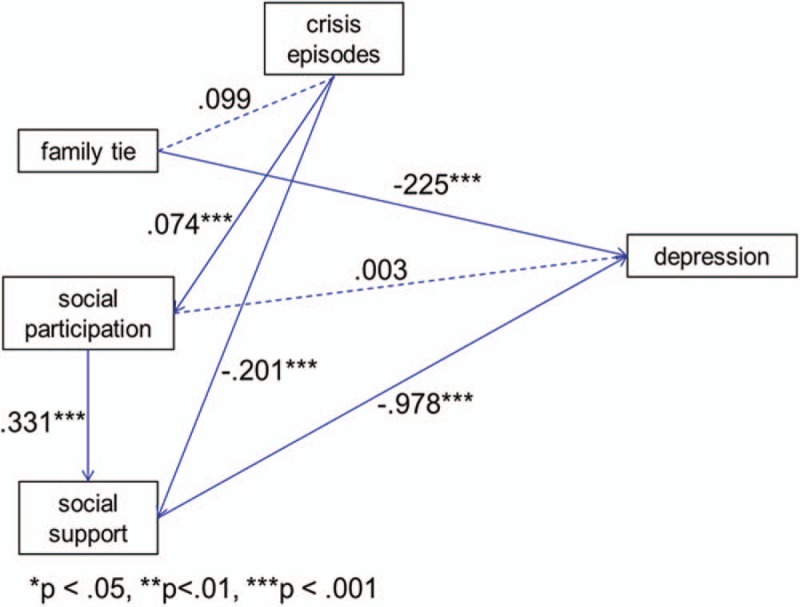
. Results of path analysis.

Figure 2 illustrates that the direct effect of crisis episodes on family ties was 0.099 (*P* > .05) and the direct effect of social participation on depression was 0.003 (*P* > .05), which did not reach the significance level. This demonstrates that the parameter estimations of these 2 paths did not have statistical significance. Family ties had a significant negative predictive power on depression (β = −0.225, *P* < .001), as did social support (β = −0.978, *P* < .001). This indicates that participants with more family ties or greater social support have a lower depression status.

Table 4 presents the regression analysis of crisis episodes and protective factors in depression. Because crisis episodes did not exhibit significant effects on family ties, family ties did not fulfill the criterion to mediate the effect of crisis episodes on depression. Social participation and depression did not exhibit significant effects. Therefore, social participation also did not fulfill the criterion to mediate the effect of crisis episodes on depression. The predictive power of crisis episodes on depression through social support was 0.197 (−0.201 × −0.978 = 0.197, *P* < .001), which was significant. This indicates that social support plays a complete mediating role between crisis episodes and depression. The predictive power of crisis episodes on depression through social participation was −0.324 (−0.331 × −0.978 = −0.324, *P* < .001), which was significant. This reveals that social participation plays a mediating role between crisis episodes and depression.

**Table 4:**
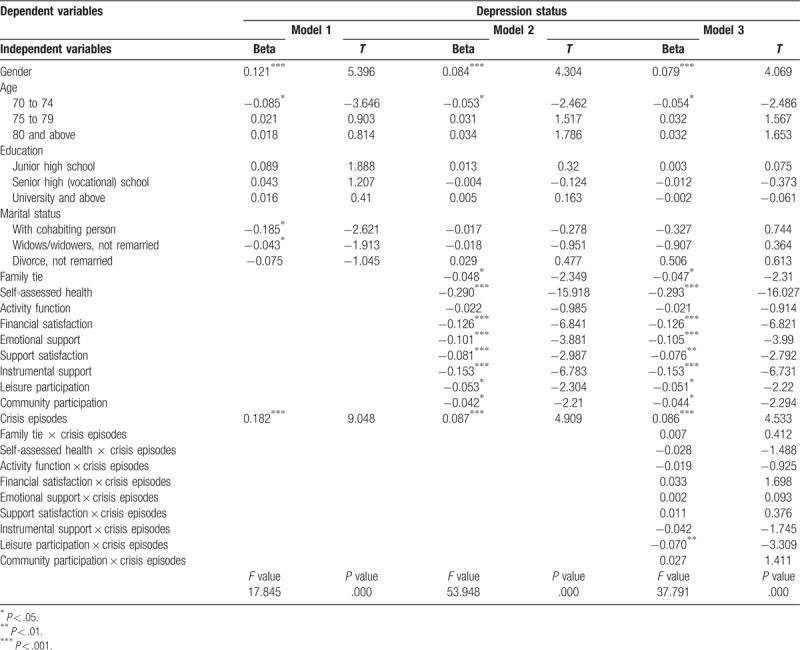
Regression analysis of crisis episodes and protective factors in depression.

## 4 Discussion

### 4.1 Clinical implications

The results of the study provide rich empirical research data for the academia; fill the gap in process study results on crisis events, protective factors, and resilience in elderly people in Taiwan; and serve as reference for other researchers to conduct similar studies. In practice, the results could be applied as a reference to strengthen effective protective factors, regardless of whether the effort is directed toward elderly people or their family members and caregivers. This could improve resilience in elderly people, enable them to cope with dilemmas rapidly and effectively when faced with crisis episodes, and help them restore their health and a satisfactory life. In particular, the Taiwanese government is currently aggressively promoting long-term care policies. However, progress is limited by a severe deficiency in funds and care workforce. Therefore, the findings of this study can assist the government in formulating effective elderly welfare policies, optimizing the use of limited funds, and effectively solving the problem of workforce shortage.

Aging usually results in the degeneration of physical function, overall disability, and multiple chronic diseases, which is compounded by a reduction in social function and role, making elderly people prone to depressive emotions.^[17]^ Depression is usually considered as a normal part of aging, which is why its multiple negative effects are often being overlooked.^[18]^ Elderly people with depressive symptoms may experience physical impairment.^[19]^ In addition, depression can cause elderly people to question the meaning of life, lose interest, and be disappointed in the future, sometimes leading to suicidal ideation.^[20]^ Several studies have reported that depression is a major risk factor for suicide in elderly people.^[21–24]^ Depression is the most common psychological problem encountered by elderly people, and it has critical effects on their emotions, physical health, social function, and suicide rates.^[25–27]^ Therefore, in this study, the reduction of depression was considered a positive adaptation index for crisis episodes in the elderly population.

When this generation of elderly Taiwanese people was young, men were traditionally portrayed as breadwinners, whereas women were relegated to managing the household. In such a cultural context, women rarely went out to work or participate in formal social activities.^[28,29]^ In addition, the average life expectancy of women is higher than that of men, and they marry at a younger age. Therefore, they are more likely to become widows and to live alone, encountering financial difficulties^[25,30]^ and becoming primary caregivers when family members are sick. Owing to the aforementioned reasons and the fact that women are more prone to emotion internalization, if lacking appropriate catharsis channels, they are more prone to depressive emotions.^[31,32]^ Our study also revealed that women are more prone to depression, which is consistent with the results of prior local and overseas studies.^[28,31–33]^

Previous studies have probed single crisis episodes, for example, a previous study^[34]^ reported that people who have been abused may have low adaptation skills. Even so, approximately 22.1% of people who were abused could effectively demonstrate resilience and fulfill criteria for successful adaptation. However, elderly people encounter diverse crisis episodes, including biological, psychological, and social crises, and reasons for their maladaptation are usually not limited to a single experience or event.^[35,36]^ Based on existing studies, common crisis episodes in elderly people include bereavement, chronic diseases, retirement, restricted daily activities, residence migration, economic safety threat, loneliness, and end of life.^[37,38]^ These various stress scenarios can sometimes occur together. Previous studies on resilience have mostly focused on children and adolescents,^[3,35,39]^ examining how children and adolescents in high-risk populations were able to maintain psychological health during trauma. Few studies have probed how resilience, that is, protective factors both from personal characteristics and environmental resources, help elderly people alleviate the effect of multiple crisis episodes.^[3,4,40]^

Protective factors can be classified into internal and external aspects. Internal protective factors focus on individual characteristics or abilities and emphasize the fact that resilience is an intrinsic protective mechanism that can be used to cope with environmental stress or potential traumatic events.^[41]^ External protective factors refer to those that can promote individual resilience in family and social systems, such as family ties, social networks, social participation, and social support,^[42]^ which can in turn alleviate the effect of unfavorable events and help achieve functional adaptation. Some studies have described 3 categories of protective factors as follows: personality traits, including autonomy, self-esteem, and positive social orientation; family support, that is cohesive, warm, and without disputes; and external resources, which can provide encouragement and increase the ability of the person to cope with adversity.^[35,42]^

According to Eshel et al,^[43]^ protective factors are the most basic constituent factors for individual resilience. Eshel et al^[43]^ proved the correlation between high resilience and low depressive symptoms in elderly people and considered resilience as a protective factor against depressive symptoms in this population. Protective factors not only help maintain psychological health and improve the coping strategies of elderly people when faced with crisis episodes but also help reduce the burden of caregivers. The protective factors investigated in this study include a person's resources, such as their physical health, financial capabilities, and activities of daily living; family ties; social participation; and social support.

The assessment standards for positive adaptation of resilience vary considerably in different research fields. For example, in Masten et al, psychological and social abilities were used as adaptation markers, whereas academic achievements,^[44]^ behavioral performance, and the ability to have good relationships with peers were used as the basis for determining adaptation quality.^[45]^ When examining the psychological health of elderly people, the most common problems encountered are depressive symptoms.^[46]^ The prevalence of depression in elderly people is higher than that in young populations. Among the elderly population in Japan, the prevalence of depressive symptoms is approximately 19.8% to 33.5%, and it is approximately 15.2% to 63% in South Korea.^[29,47,48]^ Based on data from the 2015TLSA conducted by the Health Promotion Administration of the Ministry of Health and Welfare, in which the shortened 11-question Iowa CES-D scale was used to measure the depression status of elderly people in Taiwan^[15]^ using 10 points as the cutoff value for depression (10 points and above: depression present; less than 10 points: depression absent), the proportion of elderly people with depression increased with age, with 17.3% of people aged 75 years and above experiencing depression.

Previous studies have also reported that elderly people with a higher educational level gave more importance to interpersonal relationships and maintaining physical and mental health^[49]^ and thus experienced better psychological health and adaptation to life.^[50]^ Another study indicated that families with lower educational levels often had lower financial capabilities and more severe depression.^[51]^ The present study proved that elderly people with a senior high school (vocational school) degree have a higher life satisfaction than those with an elementary school educational level or below. In addition, people with higher educational levels are more likely to accept new things and are able to use their knowledge and skills in a flexible way to solve problems when faced with difficulties and setbacks,^[50]^ which reduces their feeling of helplessness and depression.

To an elderly person, the death of a spouse is not only the loss of companionship and care in daily life but, more importantly, it results in changes in family resources, living environment, relationships with friends and family, and daily routine. Therefore, the death of a spouse is considered the main source of stress for elderly people.^[52]^ Elderly people with spouses have better adaptive capabilities than those without spouses, and the latter are more likely to develop negative emotions.^[53]^ Our study also indicated that elderly people who lost their spouses but did not remarry were more prone to depression. Therefore, the companionship of family members and friends has significance in terms of life adaptation among elderly widows or widowers.^[50]^ This is particularly true in Taiwanese society, which revolves around family. People who do not live with their parents are encouraged to visit and accompany them often.

Zhang et al^[50]^ revealed that elderly people with a high resilience better adapt when faced with crisis episodes. They experience less depressive emotions than their counterparts with low resilience.^[50]^ In addition, adaptive capabilities significantly vary according to sex, marital status, and educational level, which is consistent with the aforementioned results discussed in the present study. Furthermore, resilience is an intrinsic protective factor that can help people maintain good adaptability when they encounter crisis episodes.^[54]^ In this study, we discovered that self-perceived health and financial satisfaction were crucial factors reducing depression in elderly people. Self-perceived health is an overall assessment of one's physical health. Although it is a subjective assessment, it is positively correlated with actual health status.^[26,48]^ Elderly women with poor physical function often experience more negative emotions, which affects their self-concept and attitude toward life.^[55]^ Therefore, self-perceived health not only reflects current health status but also predicts depression. Elderly people with better financial status have better nutritional status, healthier bodies, more joyful emotions, and more harmonious interpersonal relationships.^[31]^ Previous studies have also reported that elderly people with good financial status are more able to enjoy life, make friends, and develop their own interests. They can maintain a better psychological status and have higher adaptability.^[50]^ Therefore, these people are less likely to develop psychological depression. From this, we can deduct that the financial status of elderly people not only affects their physical health but also their quality of life. Establishing laws to ensure the financial security of elderly people is key to their well-being.

Regarding family resilience, our study revealed that elderly people who lived with their spouses or had more children living with them were less likely to develop depressive emotions. Kim et al^[7]^ concluded that family resilience is a major factor that increases positive adaptation in elderly patients with dementia. Elderly people who live alone are prone to feelings of loneliness and isolation, which worsens their depression.^[56]^ Zhang et al^[50]^ reported that elderly people who are closer to their children maintain a better psychological state. Hsu^[57]^ pointed out that the concept of successful aging includes staying with one's family and the acceptance of emotional care among other criteria. The companionship of spouses or family members can have a considerable effect on the psychological health status of elderly people.^[56]^ When grandparents in extended families help look after the children, the fertility rate of women in the family is higher, and the employment ratio is also higher.^[58,59]^ Additionally, young people can assist in caring for elderly people in the family, particularly female relatives who have lost their spouses in old age,^[60]^ who require substantial care and companionship. Therefore, the extended family arrangement is a direction that must be actively explored.

Human beings are social animals. With the exception of those few who prefer solidarity, the scope and quality of social interactions and level of social support affect humans’ emotions.^[61]^ The lifestyle needs of elderly people are not only dependent on family support but also community care. Based on stress theory in cognitive research, people evaluate the effects of stress when faced with crisis episodes and search for usable resources around them to cope with stressful events.^[62]^ People with more social support tend to adopt a more positive perspective to interpret stressful events. In addition, they possess more resources to handle the effect of stressful events. Therefore, social support is often considered a coping strategy for handling stress in that it helps people reduce or buffer the negative effects of crisis episodes, reduce depressive symptoms, enhance quality of life, and improve life satisfaction.^[30,63]^ Previous studies have also indicated that people with more social support mobilize more easily different coping strategies to alleviate the effect of mental stress on depressive symptoms.^[63]^ Elderly people who lack social support are more prone to isolation, loneliness, and depressive symptoms.^[64]^ Some studies have even demonstrated that social support is an effective protective factor against chronic pain in elderly people because it can alleviate depressive symptoms caused by chronic pain.^[6]^ This study revealed that elderly people who have someone to provide care and show concern when they are feeling down and to offer them assistance when necessary, that is to say, those who have a better social support are less likely to be depressed. In addition, social support is the most direct and effective protective factor. Social participation exerts its protective function mainly by effectively increasing social support, which in turn reduces depression. Social support acts as an external defense mechanism, whereas self-resilience is an internal defense mechanism for better adaptation to crisis episodes.^[54,65]^

Old age corresponds to the time of retirement, which leads to fewer social contacts and the loss of one's social role. This situation is compounded by a large increase in free time. Therefore, elderly people need to participate in some community activities, from which they can derive dignity and happiness. In this study, we observed that elderly people who were deeply invested in leisure activities and community participation were less likely to become depressed. Pressman et al^[66]^ mentioned that active participation in leisure activities can promote physical health, reduce depressive emotions, and increase positive emotions. Meeks et al^[67]^ examined the relationship between participation in activities and positive emotions in residents of long-term care institutions. They noted that participating in activities of interest to residents could increase positive emotions and decrease depressive symptoms. Participation in social activities can improve the trust and mutual assistance between community residents and increase the psychological health of residents.^[68]^ Social participation can be used to build broad social networks and increase social resources, social capital, and social support, which are all external protective factors indispensable to elderly people.^[69]^ Actively encouraging elderly people to participate in community activities or volunteer work will not only prevent or delay physical deterioration but also reestablish their social roles and improve their self-determination.^[63,70]^ In addition, community resources around the residence, quality of public facilities, convenience of transportation, autonomy over personal finances, and companionship and encouragement from friends and family all affect the choice of leisure activities by elderly people.^[71]^ Currently, the Taiwanese government is actively promoting community meal services.

Fraser et al^[72]^ determined that protective factors could be classified as compensatory, buffering, and cumulative. Among these factors, buffering protective factors have interaction effects with crisis episodes and reduce the effect caused by crisis episodes, serving as moderators in statistics.^[73]^ The leisure activities examined in this study moderated the relationship between crisis episodes and depressive emotions. In particular, leisure activities considerably reduced the occurrence of depression in elderly people who encountered fewer crisis episodes. Regularly engaging in leisure activities can promote the physical and mental health, delay aging of physiological functions, improve self-confidence, and enhance the quality of life of elderly people.

In this study, an examination of mediating effects demonstrated that crisis episodes do not actually directly affect depressive symptoms. Instead, they maintain or reduce depressive emotions through social support. The study of Zhao et al on loneliness in the elderly population in Chinese cities revealed that social support has some mediating effects on depressive symptoms in elderly people.^[8,50]^ Protective factors are resources against environmental stress, and resilience is a dynamic process of mutual growth, influence, and balance between “crisis” and “resources.” In the resilience model proposed by Richardson,^[14]^ the protection of individual and environmental factors generated a series of psychological adjustment processes with external stress. Individuals are originally in a comfort zone. However, when encountering adversity in life, this original harmonious state is broken. If individuals do not possess sufficient protective factors to respond, they may experience a “disruption” in their psychological process. That is, individuals try to find a way out and enter the stage of “reintegration.” Individuals go back and forth between “disruption” and “reintegration” in response to an external dilemma until new ideas, attitudes, or skills are sufficient to cope with said dilemma. Our study once again confirmed the importance of protective factors when encountering crisis episodes. From a preventive standpoint, government departments, scholars and experts on elderly welfare, caregivers, and elderly people themselves must intend to strengthen the social support in this population to help them cope with external crisis episodes.

### 4.2 Methodological considerations

This study mainly examined the effects of various protective factors on alleviating depression in elderly people who experienced crisis episodes. However, various protective factors may affect one another. An example is the willingness and ability of elderly people to participate in community activities, which may be affected by their physical health and level of restriction in daily activities^[74,75]^ and even by their educational level and financial situation.^[50]^ Participation in community activities further affects the quality and quantity of social support and has complex interactions with depression in elderly people. Moreover, a long-term cohort study database was used for secondary data analysis in this study. Questionnaire items were designed for general use purpose, which means it was not completely suited to the topic under study. Therefore, the choice of study variables was limited by the original database. Finally, this study only included a cross-sectional examination of elderly people in Taiwan. The results must be interpreted with caution, and causal relationships must be inferred carefully.

## 5 Conclusion

The results revealed that the higher the number of crisis episodes faced by elderly people, the more severe their depressive symptoms. However, the effect of crisis episodes on depression is not direct. Instead, it affects the operation of social support, which in turn reduces depressive emotions. Elderly people who engage in leisure activities and community participation are less likely to develop depression. However, the protective effect of social participation is mainly derived from social support. This observation highlights the importance of social support in the elderly population. Strengthening effective protective factors can improve the resilience of elderly people and enable them to cope with dilemmas rapidly and effectively when faced with crisis episodes as well as to restore their health status and live a satisfactory life.

## Acknowledgments

The authors thank the Sunflower Statistical Consulting Company, Kaohsiung, Taiwan for statistical advice.

## Author contributions

Yang-Tzu Li cenducted the study and dafted the manuscript. Yang-Tzu LT and Tao-Hsin Tung participated in the design of the study and performed statistical analyses. All of the authors read and approved the final manscript.
